# Rainfall increasing offsets the negative effects of nighttime warming on GHGs and wheat yield in North China Plain

**DOI:** 10.1038/s41598-021-86034-3

**Published:** 2021-03-22

**Authors:** Yaojun Zhang, Wenkai Shou, Carmelo Maucieri, Feng Lin

**Affiliations:** 1 International Joint Research Laboratory for Global Change Ecology, School of Life Sciences, Henan University, Kaifeng, 475004 Henan China; 2 Institute of Applied Ecology, Chinese Academy of Sciences, 72 Wenhua Road, Shenyang, 110016 Liaoning China; 3 College of Forestry, Henan Agricultural University, 63 Agriculture Road, Zhengzhou, 450002 Henan China; 4 Department of Agronomy, Food, Natural Resources, Animals and Environment–DAFNAE, University of Padua, Agripolis Campus, Viale dell’Università 16, 35020 Legnaro, PD Italy; 5 School of Environmental Engineering, Nanjing Institute of Technology, Nanjing, 210000 Jiangsu China

**Keywords:** Biogeochemistry, Climate sciences, Environmental sciences

## Abstract

The effects of nighttime warming and rainfall increasing on crop productivity and soil greenhouse gas emissions are few studied. This study was conducted with a field experiment to investigate the effects of nighttime warming, rainfall increasing and their interaction on wheat grain yield, methane (CH_4_) and nitrous oxide (N_2_O) emissions during a winter wheat growing season in the North China Plain (NCP). The results showed that nighttime warming and rainfall increasing significantly altered soil temperature and moisture, and thus the CH_4_ and N_2_O emissions from the soil. Nighttime warming significantly promoted soil CH_4_ uptake by 21.2% and increased soil N_2_O emissions by 22.4%. Rainfall increasing stimulated soil N_2_O emissions by 15.7% but decreased soil CH_4_ uptake by 18.6%. Nighttime warming significantly decreased wheat yield by 5.5%, while rainfall increasing enhanced wheat yield by 4.0%. The results indicate that the positive effect of nighttime warming on CH_4_ uptake and negative effect on wheat yield can be offset by rainfall increasing in the NCP. Generally, rainfall increasing significantly raised the global warming potential and greenhouse gas intensity induced by CH_4_ and N_2_O emissions. Overall, this study improves our understanding of agroecosystem C and N cycling in response to nighttime warming and rainfall increasing under future climate change.

Climate change, mainly induced by greenhouse gas emissions (GHGs), is expected to increase temperature and alter rainfall pattern worldwide in the future^1,2^. Methane (CH_4_) and nitrous oxide (N_2_O) emissions from soils are two important GHGs contributing to global warming and are in turn virtually affected by climate change^3^. Agricultural soil is a major contributor to CH_4_ and N_2_O emissions^4,5^. Therefore, it is crucial to have a comprehensive understanding of feedback between CH_4_ and N_2_O emissions from agricultural soil and the ongoing climate change crisis.

The global annual mean air temperature is predicted to increase by 1.0–1.7 °C by 2050, which could have a profound influence on crop growth and GHGs emissions from agricultural soils^6,7^. Most studies on GHGs emissions response to warming have concentrated on grassland, forest and tundra, and the results have shown inconsistent responses of CH_4_ and N_2_O emissions to warming from positive^8,9^, to negative^10,11^ and no change^12,13^. Meanwhile, studies examining CH_4_ and N_2_O emissions response to warming are still lacking in agricultural field, and they mainly focused on the daily mean temperature increasing^9^. However, long-term datasets and global change models have demonstrated that climate warming presents asymmetry, with nighttime temperature increasing more rapidly than daytime temperature^14,15^. To our knowledge, field studies examining CH_4_ and N_2_O emissions response to nighttime warming rarely enlist for agricultural soils.

The North China Plain (NCP) is one of the most important agricultural regions in China and the production of winter wheat (*Triticum aestivum* L.) accounts for approximately 70% of total wheat production in China^16^. Winter wheat is sensitive to changes in temperature and rainfall derived from climate change^17^. The mean air temperature in the NCP is predicted to increase approximately 1.5 °C by 2050, mainly caused by the increase in nighttime temperature during winter and spring seasons, which will give rise to profound impact on wheat production and the soil GHGs emissions^16,18,19^. Furthermore, rainfall is predicted to increase during winter wheat growing season in this region^17^. Additionally, soil temperature and moisture are two key drivers influencing CH_4_ and N_2_O emissions^8^. Unfortunately, few studies explored the CH_4_ and N_2_O emissions from agricultural soil as response to rainfall increasing and the combined effects with nighttime warming. Thus, understanding the winter wheat productivity and GHGs emissions response to nighttime warming and increased rainfall for predicting the climate-driven changes in agroecosystem is necessary.

In this study, we carried out a field plot experiment to examine the effects of nighttime warming and rainfall increasing on CH_4_ and N_2_O emissions and wheat yield. We hypothesized that: (1) both nighttime warming and increased rainfall could stimulate CH_4_ and N_2_O fluxes, (2) nighttime warming could decrease wheat yield while rainfall increasing could alleviate the negative effect.

## Results

### Rainfall, soil moisture, and soil temperature

**Figure 1:**
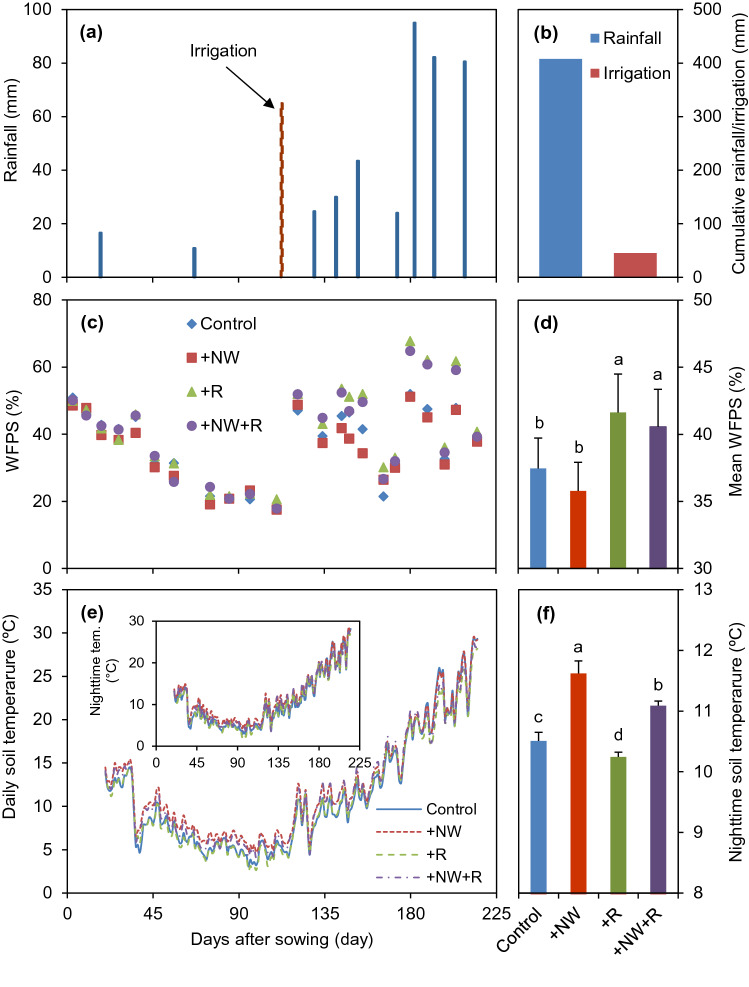
Dynamic changes and mean account of rainfall (**a**,**b**), soil moisture (WFPS, **c**,**d**) and soil temperature (**e**,**f**) during observation periods. The bars indicate the standard error of the means (± SE). Different letters indicate significant differences among treatments (Fisher's LSD Tukey HSD test at *p* < 0.05).

The seasonal changes in rainfall, soil moisture and soil temperature are shown in Fig. 1. Total rainfall amount during wheat growing season was 408 mm with more than 87% occurring in spring (from March to May). Due to winter drought, an irrigation event was required during the wintering stage (February 11, 2019), which accounted for 11% of the seasonal total rainfall (Figs. 1a and 1b).

Soil moisture showed a prompt response to rainfall and irrigation events during the growing season (Fig. 1c). The average soil moisture content of WFPS (water filled pore space) were 37.5%, 35.8%, 41.6% and 40.6% for the control, + NW, + R, and + NW + R treatments, respectively. The + R treatment significantly increased soil moisture by 16.4% compared with + NW treatment (Fig. 1d).

Soil daily and nighttime temperature are shown in Fig. 1e,f. The nighttime soil temperatures were significantly different among treatments with average values of 11.6 °C, 11.1 °C, 10.5 °C, and 10.2 °C for the + NW, + NW + R, control, and + R treatments, respectively. On average, the warming device significantly increased the nighttime soil temperature at 10 cm depth by approximately 1.1 °C under typical environmental conditions (+ NW *vs.* Control) and 0.8 °C under higher rain simulated conditions (+ NW + R *vs.* + R).

### Soil CH_4_ emissions

**Figure 2:**
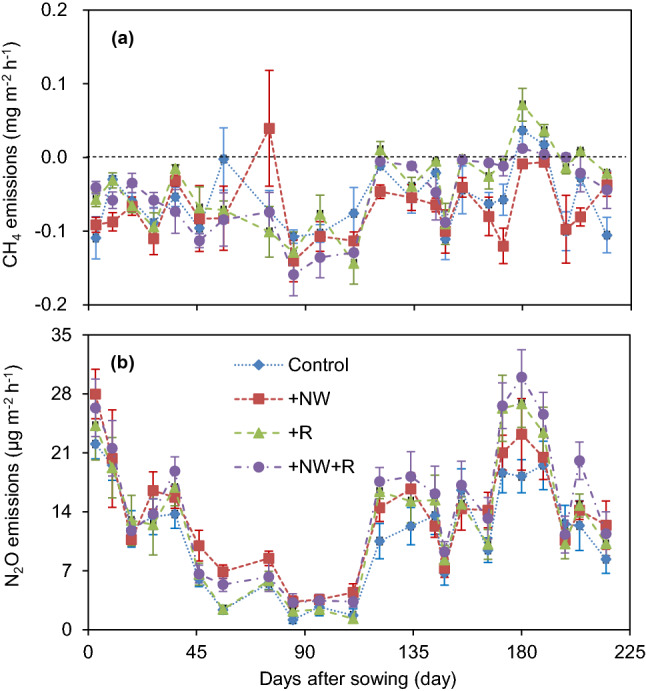
Seasonal dynamics of soil CH_4_ (**a**) and N_2_O (**b**) fluxes (mean ± SE) during observation periods.

Soil CH_4_ fluxes presented a similar pattern among the different treatments without a clear seasonal variation (Fig. 2a). Over the whole wheat growing season, CH_4_ fluxes showed almost exclusive uptake, except for some sporadic CH_4_ releases. Seasonal mean CH_4_ fluxes showed an average highest value of − 0.04 mg m^−2^ h^−1^ (ranging from − 0.14 to 0.07 mg m^−2^ h^−1^) and an average lowest value of − 0.07 mg m^−2^ h^−1^ (ranging from − 0.14 to 0.04 mg m^−2^ h^−1^), and occurring in + R and + NW treatments, respectively.

**Figure 3:**
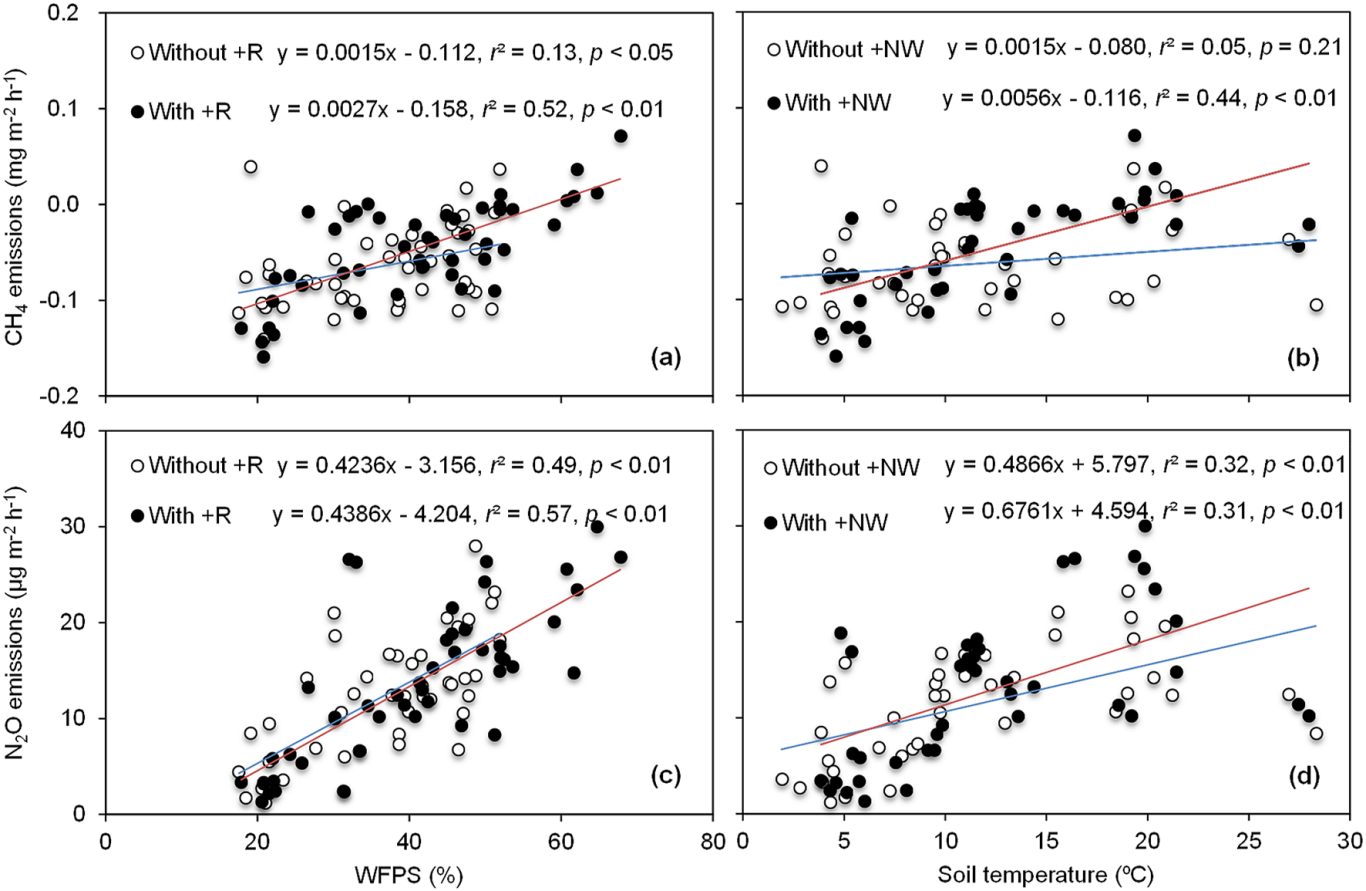
CH_4_ (**a**,**b**) and N_2_O (**c**,**d**) emissions related tolinear relationship with soil moisture (WFPS) and or soil temperature.

**Figure 4:**
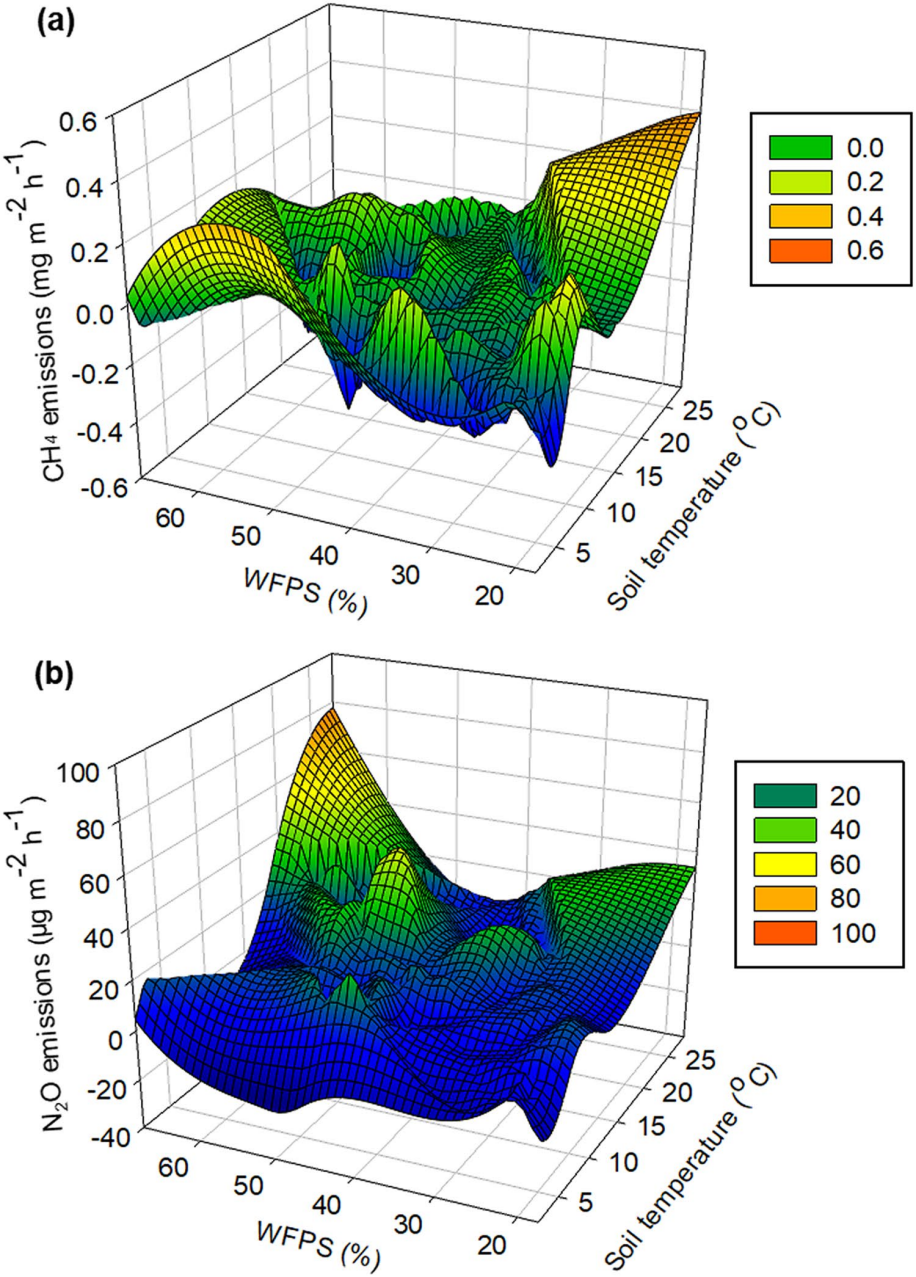
CH_4_ (**a**) and N_2_O (**b**) emissions responded response to both soil moisture and temperature during wheat growing season.

Soil CH_4_ fluxes were positively associated with soil moisture and showed more sensitive response to variation in rainfall (Fig. 3a). However, soil CH_4_ fluxes were temperature-dependent and exhibited generally higher with nighttime warming (Fig. 3b). Generally, CH_4_ fluxes primarily dependent on soil temperature and can neglect the soil moisture effects in the present study (Fig. 4a).

Over the whole wheat growing season, the cumulative CH_4_ emission was significantly affected by the nighttime warming and rainfall increasing but not their interaction (Table 1). Compared with the control, + R treatment decreased soil CH_4_ uptake by 18.6% and + NW promoted soil CH_4_ uptake by 21.2%.

**Table 1:** Grain yield, cumulative CH_4_ and N_2_O emissions, GWP and GHGI (mean ± SE) in winter wheat growing season. Different letters within each parameter indicate significant differences among treatments (Tukey HSD testFisher's LSD test at *p* < 0.05).

	Yield	CH_4_ emission	N_2_O emission	GWP_100-year_	GHGI
	kg ha^−1^	kg ha^−1^	kg ha^−1^	kg CO_2_ (eq) ha^−1^	kg CO_2_ (eq) kg^−1^
Control	4171.56 ± 48.37 ba	− 2.87 ± 0.11 ab	0.53 ± 0.02 cb	58.95 ± 6.04 bc	0.014 ± 0.002 b
+ NW	3943.66 ± 41.11 cb	− 3.48 ± 0.19 b	0.64 ± 0.03 ab	73.35 ± 5.68 bc	0.019 ± 0.001 ab
+ R	4339.70 ± 35.59 a	− 2.34 ± 0.34 a	0.61 ± 0.03 ab	101.67 ± 12.60 ab	0.023 ± 0.003 a
+ NW + R	4274.05 ± 51.38 ab	− 2.89 ± 0.27 ab	0.69 ± 0.02 a	108.49 ± 5.48 a	0.025 ± 0.002 a
ANOVA					
NW	**0.015**	**0.035**	**0.002**	0.211	0.337
R	** < 0.001**	**0.040**	**0.023**	** < 0.001**	**0.011**
NW × R	0.141	0.915	0.534	0.645	0.337

*GWP* Global warming potential, *GHGI* Greenhouse gas intensity.

Bold values indicate statistical significance with *p* value (*p* < 0.05).

### Soil N_2_O emissions

Soil N_2_O fluxes showed a typical pattern with a clear seasonal variation and seemed to be affected by soil moisture and temperature (Figs. 1c,e and 2b). Substantial N_2_O emissions occurred in the seedling stage and after jointing stage with warm temperature, while N_2_O fluxes were relatively lower during the wintering period with low temperature. Several N_2_O flux peaks were mainly observed following with rainfall or irrigation. The highest N_2_O flux emission rate came from the + NW + R treatment with a value of 30.0 μg m^−2^ h^−1^, and the lowest was from the control treatment with a value of 1.2 μg m^−2^ h^−1^, which is an approximately 25-fold difference.

Although N_2_O fluxes were depended on soil moisture, rainfall increase had no effect on N_2_O fluxes (Fig. 3c). However, nighttime warming exhibited a strong effect on soil N_2_O emissions, where soil N_2_O fluxes showed more sensitive response to the increased temperature (Fig. 3d). Generally, nighttime warming and rainfall increasing showed a comprehensive effects on N_2_O fluxes, with N_2_O fluxes increased with soil temperature companied with soil moisture (Fig. 4b).

Over the whole wheat growing season, the cumulative N_2_O emission was significantly affected by the nighttime warming and rainfall increasing but not their interaction (Table 1). In general, compared with control, + NW, and + NW + R treatments significantly increased soil N_2_O emissions by 22.4% and 32.1%, respectively.

### Wheat yield, GWP and GHGI

Nighttime warming and rainfall increasing showed significant effects on wheat grain yield but not their interaction (Table 1). The increase of nighttime warming significantly decreased (− 7.5%) the wheat yield than other treatments (4261.8 kg ha^−1^). In a context with higher rain quantity the negative effect of nighttime warming was annulled showing the same yield obtained under typical environmental conditions (no significant differences among + R, + NW + R and Control).

The GWP induced by CH_4_ and N_2_O emissions was significantly increased than Control by the rainfall increasing under both conditions, with (+ NW + R; + 84.1%) and without (+ R; + 72.5%) nighttime warming (Table 1).

Rainfall increasing also significantly increased the GHGI (by 65.7%) and nighttime warming aggravated its effect (by 79.5%) relative to the control (0.014 kg CO_2_ equivalent kg^−1^).

### Microbial biomass carbon and nitrogen

**Figure 5:**
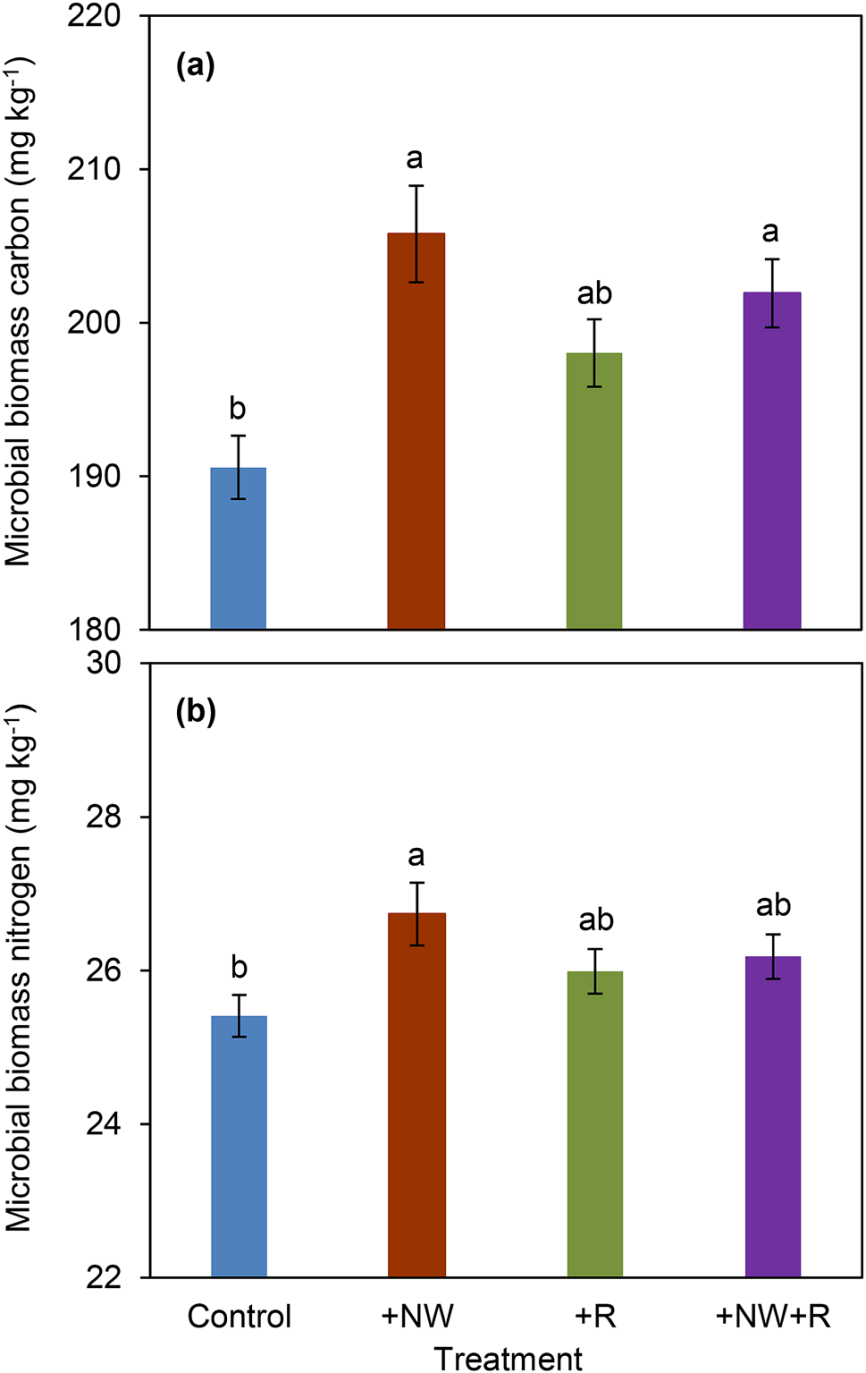
Effects of nighttime warming and rainfall increasing on soil microbial carbon (**a**) and nitrogen (**b**). Different letters indicate significant differences among treatments (Fisher's LSDTukey HSD test at *p* < 0.05).

Rainfall increasing and nighttime warming significantly affected microbial biomass carbon (MBC) and nitrogen (MBN) in the soil (Fig. 5). Compared with control treatment, + NW and + NW + R significantly increased MBC by 8.0% and 5.9%, respectively. The rainfall increasing alone (+ R) did not show significant differences with the MBC measured under typical environmental conditions (Control) (Fig. 5a). Although rainfall increasing had no effect on MBN, nighttime warming enhanced MBN content by 5.2% in soil than Control. It is worth noting that the interactive of nighttime warming and rainfall increasing had significant effects on MBC, but had no effect on MBN (Table 2).

**Table 2:** Two-way ANOVA (Tukey HSD test) of nighttime warming (NW) and rainfall increasing (R) on microbial biomass carbon (MBC) and nitrogen (MBN) at wheat harvest.

*Source*	*df*	MBC			MBN		
		*SS*	*F*	*P*	*SS*	*F*	*P*
NW	1	727.330	15.183	**0.001**	4.613	5.635	**0.025**
R	1	25.740	0.537	0.47	0.001	0.001	0.972
NW × R	1	256.738	5.359	**0.028**	2.576	3.147	0.087

*GWP* Global warming potential, *GHGI* Greenhouse gas intensity.

Bold values indicate statistical significance with *p* value (*p* < 0.05).

## Discussion

Temperature and soil moisture are two crucial factors influencing CH_4_ emissions from soils^20,21^. Enhanced CH_4_ emissions from soils with rising temperature have been well reported in previous studies, and most of the studies by using free-air CO_2_ enrichment system or open-top chambers^21–23^. The positive effect of warming enhanced CH_4_ emissions was mainly due to the canopy warming could increase soil temperature, thus enhanced soil organic matter decomposition providing abundant substrate for methanogen producing CH_4_^9^. In contrast, our results showed that nighttime warming increased CH_4_ uptake by 21.2% during winter wheat growing season and CH_4_ emission was significantly correlated with soil temperature under nighttime warming (Table 1, Fig. 1b). Generally, the main processes of CH_4_ turnover in soil including CH_4_ production, oxidation, transportation and emission, any factor influencing the processes would affect CH_4_ flux in soil^24^. Previous studies confirmed that the stimulation of warming on CH_4_ uptake was mainly attribute to its effects on the abundance of methanotrophs^25,26^. In addition, soil temperature might be the primary driving factor for CH_4_ emission/uptake in dryland when soil moisture is relatively low^27^. Soil aerobics and gas diffusivity increased when the soil water content decreased, thus CH_4_ consumption enhanced^28,29^. However, when soil moisture is too low, CH_4_ diffuse without restriction but microbes are physiologically stressed and its activity, including methanotrophs, is reduced, which reduces the CH_4_ uptake^30^. In the present study, soil moisture was mostly below 60% WFPS, and especially less than 40% during the winter growing season (Fig. 1c). Nighttime warming reduced soil moisture (Fig. 1d), thus could be the main reason resulting in most CH_4_ uptake by the soil.

Relative to temperature, soil moisture is a more vital factor influencing CH_4_ emissions in soil^28^. In the present study, rainfall increasing by 30% reduced the CH_4_ uptake by 18.5%. Consistent with our study, Guo et al. (2015) ^31^ reported that increasing rainfall resulted in an 18.2% reduction in the CH_4_ uptake rate. The slope of simulated regression was higher for the treatments with increased rainfall than the controls, suggesting rainfall increasing had intensified the response of CH_4_ emissions to soil moisture (Fig. 3a). Actually, the + R treatments with rainfall increasing give rise to a higher soil moisture. Although the confidence interval with + R greater than that without + R, the regressions could partially explain the results. High soil moisture might decrease soil O_2_ content, thus provided an anaerobic environment favoring for soil CH_4_ production and emission^32–34^. Interestingly, our results found that the interaction between nighttime warming and rainfall increasing had no effect on CH_4_ emission, indicating that rainfall increasing might offset the negative effect of warming on CH_4_ emission. Additionally, higher MBC content was detected under both nighttime warming and rainfall increasing, which may also contribute to the higher CH_4_ consumption at + NW and + R treatments. Generally, microbial activity mainly depends on soil temperature and moisture, thus affecting soil CH_4_ flux.

In our study, nighttime warming significantly enhanced cumulative N_2_O emissions which were positively correlated with soil temperature for the control and nighttime warming treatments (Table 1, Fig. 3d). Our results were consistent with several previous studies^3,10,35^. Smith et al.^36^ mentioned that the N_2_O fluxes were expected to increase with rising temperature. Denitrification, main process of N_2_O formation in the soil, could be enhanced by higher aerobic respiration and higher oxygen consumption under warming^37^. Furthermore, many studies reported that warming accelerated soil organic matter mineralization, which increased the substrate for N_2_O production^38,39^. In contrast, Liu et al.^11^ reported that warming significantly reduced N_2_O fluxes in northeast China, due to the decreased soil water content caused by higher temperature. The optimal WFPS of N_2_O emissions was in the range of 70–80%. While in our study, relative sufficient rainfall occurred during wheat growing season (408 mm) and irrigation event occurred in the winter drought stage, which led to the higher N_2_O emission under nighttime warming. Overall, the effect of warming on N_2_O emission will depend on the offsets between the positive and negative effects of warming on N_2_O production processes.

In this study, the N_2_O flux peaks mainly followed rainfall and irrigation, indicating that soil moisture primarily regulates the spatial and seasonal variability of N_2_O emission. Generally, the increase in soil water content following dry conditions stimulated microbial turnover and N_2_O flux^40^. N_2_O emissions were positively correlated with soil moisture in our study, the regression slope of with + R was higher than that of without + R, indicating that rainfall increasing had a stimulate effect on N_2_O emissions (Fig. 3c). Similar to nighttime warming, rainfall increasing significantly enhanced cumulative N_2_O emission and further enhanced cumulative N_2_O emission under nighttime warming. It is probably due to the higher temperature and soil moisture stimulate the microbial activity and denitrification that caused the increase of N_2_O emissions. The MBN contents were higher under both nighttime warming and rainfall increasing treatments, which could also contribute to the N_2_O discharge.

Our results showed that nighttime warming significantly decreased wheat yields relative to the control. Similarly, previous studies reported that climatic warming might cause a substantial loss in Chinese wheat yields^41,42^. In contrast, some other studies suggested that nighttime warming benefit winter wheat production in China^18,19^. They attribute the positive effects of nighttime warming on winter wheat yields to more suitable temperature condition for winter wheat growth under warming treatment.

Instead, rainfall increasing offset the negative effect of nighttime warming on wheat yield showing no significant differences with actual typical environmental conditions. To our knowledge, soil temperature and moisture are two main keys influencing plant productivity. Soil water availability will decrease due to enhanced evapotranspiration under climate warming, thus limiting microbial activity, nutrient availability and plant growth. Chavas et al.^43^, using a simulation model under future climate scenario, suggested that winter wheat productivity increased significantly with the increase of precipitation in the North China Plain. However, Song et al.^17^ reported that excess precipitation decreased winter wheat yields due to the induced diseases such as Fusarium head blight.

It was known that both temperature and soil moisture contribute to soil microbial growth, communities, activities and processes, which affected CH_4_ and N_2_O fluxes^34,44,45^. However, this study did not consider the microbial processes of nitrification and denitrification for N_2_O production and the methanogenic and methanotrophic community associate with CH_4_ fluxes. Thus, future studies should be focused on the microbiological level in farmland systems to clarify the effects of nighttime warming and increasing precipitation on CH_4_ and N_2_O fluxes. In addition, our experiment was conducted for only one winter wheat growing season. The long-term effect of nighttime warming and increased rainfall on CH_4_ and N_2_O fluxes remains unclear. It is necessary to assess the impact of warming and precipitation on CH_4_ and N_2_O emissions at large time-scale.

## Conclusions

The present study showed that nighttime warming and rainfall increasing could affect wheat productivity and greenhouse gas emissions in the NCP. Nighttime warming significantly increased CH_4_ uptake and N_2_O emissions, but decreased wheat grain yield. Rainfall increasing reduced CH_4_ uptake, but enhanced N_2_O emissions and wheat grain yield. Although nighttime warming and rainfall increasing showed significant influence on wheat productivity and greenhouse gas emissions, the interaction between them had no effects. In general, the present study give us a better understanding of the agroecosystem C and N cycling in response to nighttime warming and rainfall increasing under future climate change scenarios. Furthermore, the future work is necessary to assess the impact of warming and precipitation on CH_4_ and N_2_O emissions at a large time-scale.

## Materials and methods

### Study site description

The experiment was carried out during the winter wheat growing season (from October 2018 to May 2019) in a wheat–maize rotation in a field located in the periphery of Anhui Science and Technology University (32° 86′ N, 117° 4′ E), south part of the NCP. This region has a sub-tropical and sub-humid monsoon climate with a mean annual air temperature of 14.9 °C. Mean annual precipitation was 904.4 mm and approximately 40% of the rainfall was distributed in winter wheat growing season. The soil was classified as hydromorphic, consisting of 13% sand, 47% silt, and 40% clay. The upper soil layer (0–25 cm) had an initial pH of 6.5 (1:1, water/soil, w/w) and an average bulk density of 1.26 g cm^−3^. The soil available N was 64.6 mg kg^−1^, organic C content was 8.65 g kg^−1^, available phosphorus was 31.6 mg kg^−1^, and available potassium was 62.3 mg kg^−1^.

### Field experiments

Two factors (rain (R) and nighttime warming (NW)) at two levels (typical environment conditions and simulated changed conditions) with a completely randomized experimental design have been compared including the following treatments: 1) the typical environment conditions of rain and nighttime temperature (Control); 2) nighttime warming treatment (+ NW); 3) simulated rainfall increasing by 30% (+ R); and 4) the combination of nighttime warming and rainfall increasing (+ NW + R). Each treatment was four times replicated making a total of 16 plots. Each plot was 12 m^2^ (3 m × 4 m) and there was a 0.5 m buffer zone for preventing water and nutrients exchange between any two adjacent plots. For the nighttime warming treatment, an aluminum foil coated fabric was covering 30 cm above the surface of the wheat during nighttime. It was placed manually every day from sunset to sunrise, except for rainy and windy nights. The aluminum foil coated fabric reflected more than 96% of the direct radiation and diffused radiation to the soil surface thus making a passive nighttime warming^46^. For the rainfall increasing treatment, we sprayed 30% amount of the rainfall within two days after each rainfall event.

### CH_4_ and N_2_O emissions measurement

Field in situ CH_4_ and N_2_O fluxes were monitored from October 20, 2018 to May 20, 2019 (215 days) using closed opaque static chamber method. The details on sampling chamber design and structure were the same as reported in our previous studies^47,48^. Wheat plants were included within the static chamber. Specially-made boardwalks were established above the alley soil to minimize soil disturbance during gas flux measurements prior to initial gas sampling. Three parallel aluminum flux collars (50 cm length × 50 cm width × 15 cm height) were permanently installed (0.15 m in soil depth) near the boardwalks in each field plot. When the gas was sampled, the chamber was placed over the wheat with the rim of chamber fitted into the groove of the collar.

Gas samples were generally taken once a week except one more time after rainfall event or irrigation. Gas samples were taken at 0, 10, 20 min after chamber closure between 08:30 and 11:00 local time on each sampling day. Each gas sample was stored in a 100 ml gas sampling bag (Delin Gas Packing Co., LTD, Dalian, China), which was vacuumed by vacuum pump before gas sampling, and transported to laboratory for analyzing CH_4_ and N_2_O concentrations.

CH_4_ and N_2_O concentrations in the samples were quantified by a gas chromatograph (Agilent 7890 A, USA) equipped with an electron capture detector (ECD) and flame ionization detector (FID). The specific procedures for gas flux determination was detailed in our previous study^49^. Seasonal cumulative amounts of CH_4_ and N_2_O emissions during the observation period were calculated from the emissions between every two adjacent intervals of measurements.

### Wheat grain yield and other data measurements

Wheat yield was measured in each plot sampling five points (1 m × 1 m) in each plot at wheat maturity (30 May, 2019). Grain yield from each plot was collected in mesh bags and immediately weighted.

Soil temperature was monitored using an automatic data recorder with ibutton at a depth of 10 cm. The automatic data recorder was set to record the soil temperature every 2 h thus for calculating the mean whole day and nighttime temperature. Soil moisture was measured using a portable rod probe (MPM–160) when the soil emissions were sampled. Soil volumetric moisture was further converted into WFPS by the following equation: WFPS = [soil volumetric water content/(1 − (soil bulk density/2.65)) × 100%], where, 2.65 Mg m^−3^ was the assumed soil particle density^47^.

The microbial biomass carbon (MBC) and nitrogen (MBN) were measured at wheat harvest. About 10 g fresh soil was fumigated by ethanol-free chloroform for 4 days. The fumigated soil and 10 g of unfumigated soil were mixed with 50 ml 0.5 M K_2_SO_4_. The total extracted organic C and N contents were then analyzed. MBC and MBN contents were determined as the organic C and N difference with fumigated minus unfumigated, and the conversion coefficients for MBC and MBN were 0.45 and 0.54, respectively.

### GWP and GHGI calculation

The global warming potential (GWP) produced by CH_4_ and N_2_O emission was obtained at 100 − year time horizon using the following Eq.^50^:

1 $${\text{GWP}}\;\left( {{\text{Mg}}\;{\text{CO}}_{{2}} {\text{equivalent ha}}^{{{-}{1}}} } \right) \, = { 34 } \times {\text{ CH}}_{{4}} + { 298 } \times {\text{ N}}_{{2}} {\text{O}}$$

Greenhouse gas intensity (GHGI) is a frequently used measure of GHG emissions per unit of grain production and calculated by dividing the GWP by the wheat grain yields:

2 $${\text{GHGI}}\left( {{\text{Mg}}\;{\text{CO}}_{{2}} {\text{equivalent}}\;{\text{Mg}}\;{\text{grain}}^{{ - {1}}} } \right) \, = {\text{ GWP}}/{\text{grain}}\;{\text{yields}}$$

### Data analyses

Statistical analysis was performed with SPSS version 21.0 (SPSS Inc.). Two-way analysis of variance (ANOVA) was used for the comparison of wheat yield, CH_4_ and N_2_O emissions under nighttime warming and rainfall increasing. The treatments effect on soil moisture and temperature were evaluated using repeated ANOVA. In the case of a significant F-value, the means were compared using Tukey’s Honest Significant Difference (HSD) test at *p* < 0.05. All the data are showed as mean ± SE (n = 4). The single effect of soil moisture or temperature on CH_4_ and N_2_O emissions was evaluated through linear relationship. The 3D mesh plots for combined effect of soil moisture and temperature on CH_4_ and N_2_O emissions were represented using SigmaPlot version 12.5, from Systat Software, Inc., San Jose California USA, www.systatsoftware.com.
